# Metabolic Dysfunction-associated Steatotic Liver Disease Alters Fatty Acid Profiles in the Liver and Adipose Tissue

**DOI:** 10.1210/clinem/dgaf346

**Published:** 2025-06-12

**Authors:** Saana Palomurto, Kirsi A Virtanen, Vesa Kärjä, Ursula Schwab, Dorota Kaminska, Pirjo Käkelä, Jussi Pihlajamäki, Ville Männistö

**Affiliations:** Department of Surgery, Kuopio University Hospital, North Savo Wellbeing County, 70210 Kuopio, Finland; Faculty of Health Sciences, School of Medicine, Institute of Clinical Medicine, University of Eastern Finland, 70211 Kuopio, Finland; Turku PET Centre, Turku University Hospital, 20520 Turku, Finland; Department of Clinical Pathology, Kuopio University Hospital, North Savo Wellbeing County, 70210 Kuopio, Finland; Department of Public Health and Clinical Nutrition, School of Medicine, University of Eastern Finland, 70211 Kuopio, Finland; Department of Medicine, Endocrinology, and Clinical Nutrition, Kuopio University Hospital, North Savo Wellbeing County, 70210 Kuopio, Finland; Department of Public Health and Clinical Nutrition, School of Medicine, University of Eastern Finland, 70211 Kuopio, Finland; Department of Human Genetics, David Geffen School of Medicine at UCLA, Los Angeles, CA 90095, USA; Division of Cardiology, David Geffen School of Medicine at UCLA, Los Angeles, CA 90095, USA; Department of Surgery, Kuopio University Hospital, North Savo Wellbeing County, 70210 Kuopio, Finland; Faculty of Health Sciences, School of Medicine, Institute of Clinical Medicine, University of Eastern Finland, 70211 Kuopio, Finland; Department of Public Health and Clinical Nutrition, School of Medicine, University of Eastern Finland, 70211 Kuopio, Finland; Department of Medicine, Endocrinology, and Clinical Nutrition, Kuopio University Hospital, North Savo Wellbeing County, 70210 Kuopio, Finland; Faculty of Health Sciences, School of Medicine, Institute of Clinical Medicine, University of Eastern Finland, 70211 Kuopio, Finland; Department of Medicine, Internal Medicine, Kuopio University Hospital, North Savo Wellbeing County, 70210 Kuopio, Finland

**Keywords:** estimated enzyme activities, fatty acid metabolism, metabolic dysfunction-associated steatohepatitis, nonalcoholic fatty liver disease

## Abstract

**Context:**

The alterations in systemic fatty acid (FA) metabolism in metabolic dysfunction-associated steatotic liver disease (MASLD) remain unclear.

**Objective:**

To investigate intertissue crosstalk in FA metabolism in patients with MASLD, we compared FA profiles in the liver, serum, visceral, and subcutaneous adipose tissue of patients with severe obesity and normal liver, simple steatosis, or metabolic dysfunction-associated steatohepatitis (MASH).

**Methods:**

Preoperative serum, liver, subcutaneous, and visceral adipose tissue samples were collected during laparoscopic gastric bypass surgery from 183 patients with severe obesity (122 women, mean age 46.9 ± 9.7 years, body mass index 43.5 ± 5.7 kg/m^2^). FA composition was analyzed using gas-liquid chromatography. The Kruskal–Wallis test was used to compare the FA proportions in different tissue depots.

**Results:**

FA proportions varied more in the liver than in adipose tissue in patients with MASH. Polyunsaturated FAs (PUFA) proportions were significantly lower in the livers of patients with MASH than in those with normal livers (all adjusted *P* < .01). Conversely, dihomo-gamma-linolenic acid, adrenic acid, and arachidonic acid proportions were higher in the adipose tissues of patients with MASH (all adjusted *P* < .001).

**Conclusion:**

Patients with MASH exhibited reduced hepatic PUFA content, increased hepatic saturated FAs, and a higher n6-to-n3 PUFA ratio, whereas no clear trends were observed in adipose tissue. These findings highlight distinct differences in FA metabolism between the liver and adipose tissue in MASLD, emphasizing tissue-specific regulatory mechanisms.

Metabolic dysfunction-associated steatotic liver disease (MASLD), previously known as nonalcoholic fatty liver disease, is the most common chronic liver disease, with a global prevalence of 30% and rising (1, 2). MASLD is closely associated with obesity and metabolic disturbances. While it primarily manifests as hepatic steatosis, some individuals develop metabolic dysfunction-associated steatohepatitis (MASH), which can progress to liver cirrhosis and hepatocellular carcinoma (3, 4).

Hepatic steatosis is a hallmark of MASLD. During MASLD pathogenesis, free fatty acids are esterified to triglycerides (TGs) in the liver and stored as lipid droplets, causing steatosis. Free fatty acids are derived from adipose tissue lipolysis, de novo lipogenesis (DNL) in the liver, and dietary fatty acids (FA) absorbed from the gut (5). Adipose tissue lipolysis is the predominant source, accounting for 60% of FA flux to the liver (5). However, DNL is elevated in MASLD (6), which induces alterations in hepatic FA composition (7). The quality of FAs is also critical in the development of hepatic steatosis, as excess saturated FAs (SFAs) can induce mitochondrial dysfunction, apoptosis, and endoplasmic reticulum stress in the liver (8). Accordingly, the total SFA content in the liver is increased in patients with MASLD (9). Monounsaturated FA (MUFA) levels are elevated in MASLD, whereas polyunsaturated FA (PUFA) levels are reduced in MASLD (9, 10). Unsaturated FAs are considered less harmful to the liver than SFAs (11-15). Changes in the activity of stearoyl-CoA desaturase 1 (SCD1), delta-5 desaturase (D5D), delta-6 desaturase (D6D), and elongase have been linked to steatosis development (12, 16).

Subcutaneous adipose tissue (SAT) and visceral adipose tissue (VAT) are key endocrine organs involved in energy storage, though their functions differ. Increased VAT mass is associated with severity of liver steatosis and fibrosis (17). VAT contains more ceramides than SAT, which may mediate AT-induced insulin resistance (18). VAT is also more prone to inflammation than SAT in obesity and has a greater impact on systemic inflammation and metabolic alterations than SAT (19, 20). However, differences in lipid classes other than ceramides were not observed between the SAT and VAT (18).

Although alterations in FA metabolism in serum and tissues have been described in MASLD, the interplay between these depots in patients with MASLD is not well understood. A recent study focused on FA composition in the liver and serum samples of patients with MASLD, identifying distinct FA profiles associated with disease severity (7). However, alterations in FA composition in different stages of MASLD were not analyzed in adipose tissue. We assessed the alterations in FA metabolism in patients with MASLD by comparing the FA compositions in the serum and 3 different tissue depots (SAT, VAT, and liver) in 183 patients with severe obesity. This comprehensive approach provides a unique opportunity to explore depot-specific alterations in the FA profiles of patients with MASLD, offering novel insights into the tissue-level dynamics of lipid metabolism in the disease.

## Material and Methods

### Study Population

All patients undergoing laparoscopic Roux-en-Y gastric bypass surgery (LRYBG) at Kuopio University Hospital were recruited for the Kuopio Obesity Surgery Study, which investigated metabolic alterations associated with severe obesity and the metabolic consequences of obesity surgery. A total of 183 individuals who underwent LRYGB for the treatment of severe obesity were selected based on the availability of fasting serum samples, liver biopsies, and SAT and VAT samples. The number of samples from different depots is shown in Supplementary Fig. S1 (21). The study participants were similar with respect to age, sex, prevalence of diabetes, cholesterol-lowering medication, body mass index, and serum lipid, glucose, insulin, and alanine aminotransferase levels [Supplementary Table S1 (21)].

All patients attended an outpatient visit before surgery, during which clinical characteristics, comorbidities, and medication use were recorded. They followed a very low-calorie diet for approximately 4 weeks preoperatively. Blood samples were collected after 12 hours of fasting. Plasma glucose, insulin, and serum lipid levels were determined as previously described (22). Patatin-like phospholipase domain containing 3 (*PNPLA3*) at rs738409, transmembrane 6 superfamily 2 (*TM6SF2*) at rs58542926, and membrane-bound O-acetyltransferase 7 (*MBOAT7*) at rs641738 were genotyped using the TaqMan SNP genotyping assay (Applied Biosystems) according to the manufacturer's protocol. These variants were selected based on their well-established role in the risk of MASLD (23, 24). Written informed consent was obtained from all participants. The study was conducted in accordance with the principles of the Declaration of Helsinki and was approved by the Ethics Committee of Northern Savo Hospital District (54/2005, 104/2008, 27/2010, and 1108/2018).

### Liver Histology

Wedge biopsies of the liver were obtained during elective LRYGB using a Harmonic ultrasound scalpel. An experienced liver pathologist conducted the histological assessment based on standard MASLD grading criteria (25, 26). The diagnosis was classified into 3 distinct categories: (1) normal liver without steatosis, inflammation, ballooning, or fibrosis; (2) simple steatosis (>5% steatosis) without hepatocellular ballooning, inflammation, or fibrosis; and (3) MASH.

### Fatty Acid Analysis

Fasting serum samples were extracted with chloroform-methanol (2:1) solution, and lipid fractions (TG, cholesteryl ester, and phospholipids) were separated by solid-phase extraction with an aminopropyl column. AT samples were frozen and pulverized with liquid nitrogen, and ∼40 mg of AT was extracted with chloroform-methanol and processed similarly as serum samples (27, 28). Liver fatty acid concentrations in the TG fraction were quantified by gas-liquid chromatography on a 50 m long capillary column (Ultra 2, Agilent Technologies, Wilmington, DE, USA), with 5-cholestane as the internal standard (29). FA proportions were presented as molar percentages. Adrenic acid was not detected in the serum and liver samples, and pentadecanoic and heptadecanoic acids were not detected in the serum samples.

Enzyme activities were estimated using the product-to-precursor ratios of individual FAs as follows: SCD1 activity at a ratio of 16:1 n7/16:0 and 18:1 n7/18:0, elongase activity at a ratio of 18:0/16:0 and 18:1 n7/16:1 n7, D5D activity at a ratio of 20:4 n6/20:3 n6, and D6D activity at a ratio of 18:3 n6/18:2 n6 (16).

### Liver RNA Sequencing Analysis

Total RNA sequencing was conducted for liver samples as previously described (30). Briefly, libraries underwent paired-end sequencing (50-nucleotide reads) and subsequent read alignment. Gene-level counts were normalized via the trimmed mean of M-values method, converted to counts per million using edgeR and then subjected to log2 transformation. Expression data were corrected for technical covariates (RNA Integrity Number, percentage of uniquely aligned reads, 3′ bias). Expression data were assessed for 40 genes selected for their roles in FA and cholesterol metabolism.

### Data Analysis

**Figure 1.**
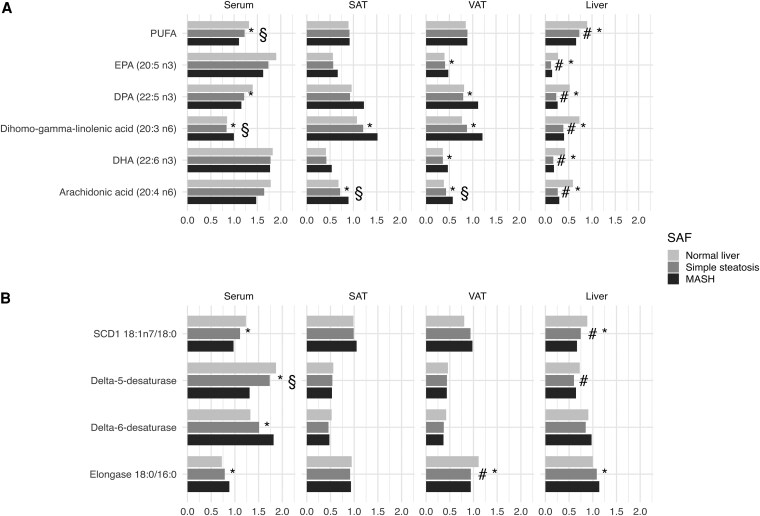
Levels of the most significant (A) polyunsaturated fatty acids and (B) estimated enzyme activities in serum, subcutaneous adipose tissue, visceral adipose tissue, and liver in patients with a normal liver, simple steatosis, and MASH. Levels of all the assessed fatty acids and estimated enzyme activities are displayed in Supplementary Fig. S1 (21) and Supplementary Table S2 (21). The values are scaled. The Kruskal–Wallis test was used to test the difference between patients with a normal liver, simple steatosis, and MASH. If the Kruskal–Wallis test yielded a significant result, the Dunn test was applied for post hoc multiple comparisons. *indicates a significant difference between individuals with a normal liver and those with MASH; ^§^between those with steatosis and MASH; and ^#^between those with normal liver and steatosis in the Dunn test. Statistical significance was defined as a false discovery rate of <0.05. Abbreviations: DHA, docosahexaenoic acid; DPA, docosapentaenoic acid; EPA, eicosapentaenoic acid; MASH, metabolic dysfunction-associated steatohepatitis; MUFA, monounsaturated fatty acid; PUFA, polyunsaturated fatty acid; SAT, subcutaneous adipose tissue; SCD1, stearoyl-CoA desaturase 1; SFA, saturated fatty acid; VAT, visceral adipose tissue.

FA proportions in each tissue depot were measured and compared among participants with a normal liver, simple steatosis, and MASH. Results are expressed as mean ± SD. A nonparametric Kruskal–Wallis analysis of variance with *P*-value adjustment, using the false discovery rate (FDR), was conducted. The FDR adjustment for the R package *P*-adjust was used to correct the *P*-values after multiple testing (31). A post hoc multiple comparison with Dunn's test was used to compare differences between the groups for FAs with significant FDR using the Kruskal–Wallis test. For the adjusted linear model, FA proportions were log-converted, and a model with FDR correction was used to adjust the *P*-values. Statistical significance was set at FDR < 0.05. Values are scaled for visualization in Fig. 1. Associations between gene expression and liver FAs were assessed using Spearman's correlation analysis, and results were corrected for multiple testing using the FDR. R (version 4.2.1); RStudio (version 2025.05.1+513) was used for the statistical analyses.

## Results

### Clinical Characteristics of the Study Population

The study included 183 patients categorized based on liver histology: 89 with a normal liver, 39 with simple steatosis, and 55 with MASH. Fasting glucose levels were higher in those with MASH than in those with simple steatosis (*P* < .05) and normal livers (*P* < .0001), while fasting insulin and serum TG levels were higher in those with MASH than in those with a normal liver (*P* < .0001 and *P* < .01, respectively) and in those with simple steatosis than in those with a normal liver (both *P* < .05). Patients with MASH had higher alanine aminotransferase levels than those with normal liver (*P* < .001) (Table 1).

**Table 1.** Clinical characteristics of the study population

	Total n = 183	Normal liver n = 89	Simple steatosis n = 39	MASH n = 55	*P*-value
Sex, female, n (%)	122 (66.7)	64 (71.9)	28 (71.8)	30 (54.5%)	.074
Age (years)	46.9 ± 9.7	45.5 ± 10.3	46.5 ± 8.5	49.4 ± 9.1	.081
BMI	43.5 ± 5.7	42.8 ± 5.8	43.9 ± 4.8	44.5 ± 6.2	.130
Fasting glucose	6.4 ± 1.8	5.9 ± 1.2	6.3 ± 1.9	7.3 ± 2.3	3.01×10^−5*a*,*b*^
Fasting insulin	19.7 ± 22.4	14.4 ± 7.3	19.2 ± 10.1	28.6 ± 37.5	7.92×10^−6*a*,*c*^
ALT	44.2 ± 31.2	36.0 ± 21.3	43.2 ± 28.6	58.0 ± 40.7	.0003*^a^*
Total cholesterol	4.2 ± 0.9	4.1 ± 0.8	4.2 ± 0.9	4.3 ± 1.1	.887
LDL-cholesterol	2.4 ± 0.8	2.4 ± 0.7	2.5 ± 0.9	2.4 ± 1.0	.904
HDL-cholesterol	1.1 ± 0.3	1.1 ± 0.3	1.0 ± 0.2	1.1 ± 0.4	.141
Triglycerides	1.5 ± 0.7	1.4 ± 0.5	1.7 ± 0.7	1.7 ± 0.8	.002^*a,c*^
Type 2 diabetes, n (%)	58 (31.7)	15 (16.9)	11 (28.2)	32 (58.2)	1.31×10^−6^
Cholesterol medication, n (%)	56 (30.6)	24 (27.0)	8 (20.5)	24 (43.6)	.035
*PNPLA3* rs738409 (%)	CC 107 (65.2), CG 47 (28.7), GG 10 (6.1)	CC 57 (72.2), CG 19 (24.1), GG 3 (3.8)	CC 20 (54.1), CG 14 (37.8), GG 3 (8.1)	CC 30 (62.5), CG 14 (29.2), GG 4 (8.3)	.361
*MBOAT7* rs641738 (%)	CC 60 (36.8), CT 73 (44.8), TT 30 (18.4)	CC 26 (32.9), CT 38 (48.1), TT 15 (19.0)	CC 15 (40.5), CT 16 99 (43.2), TT 6 (16.2)	CC 19 (40.4), CT 19 (40.4), TT 9 (19.1)	.883
*TM6SF2* rs58542926 (%)	CC 138 (84.1), CT 25 (15.2), TT 1 (0.6)	CC 69 (78.4), CT 19 (21.6), TT 0 (0.0)	CC 29 (78.4), CT 7 (18.9), TT 1 (2.7)	CC 40 (83.3), CT 8 (16.7), TT 0 (0.0)	.353

The chi-square test was used to compare categorical variables, while the Kruskal–Wallis test was used for continuous variables. If the Kruskal–Wallis test yielded a significant result, the Dunn test was applied for post hoc multiple comparisons.

A *P*-value of <.05 was considered statistically significant.

Abbreviations: ALT, alanine transferase; BMI, body mass index; HDL, high-density lipoprotein; LDL, low-density lipoprotein; MASH, metabolic dysfunction-associated steatohepatitis; *MBOAT7*, membrane-bound O-acetyltransferase 7; *PNPLA3*, patatin-like phospholipase domain containing 3; *TM6SF2*, trans-membrane 6 superfamily 2.

^*a*^Indicates a significant difference between individuals with a normal liver and those with MASH.

^*b*^Indicates a significant difference between those with steatosis and those with MASH.

^*c*^Indicates a significant difference between those with a normal liver and those with steatosis in the Dunn test.

A total of 95 serum samples, 76 SAT, 77 VAT, and 124 liver samples were obtained from patients. The overlap between the study groups is shown in Supplementary Fig. S1 (21).

### PUFAs Are Differentially Altered in Adipose Tissue and in Liver in Patients With MASH

In serum samples, the total PUFAs, n6 fatty acid, and linoleic acid (18:2 n6) proportions were significantly lower in patients with MASH than in those with normal liver (all FDR < 0.001) [Fig. 1A and Supplementary Table S1 (21)]. Dihomo-gamma-linolenic acid (20:3 n6) proportions were higher in patients with MASH than in those with a normal liver (FDR = 0.010) and in patients with MASH than in those with simple steatosis (FDR = 0.006) [Fig. 1A and Supplementary Table S2 (21)].

The sum of the different PUFAs (mole%) in SAT and VAT did not differ between the study groups. However, for individual fatty acids, dihomo-gamma-linolenic acid (20:3 n6), arachidonic acid (20:4 n6), and adrenic acid (22:4 n6) proportions in SAT and VAT were higher in patients with MASH than in those with a normal liver (all FDR < 0.001). In addition, docosapentaenoic acid (22:5 n3) proportions in VAT were higher in patients with MASH than in those with normal liver (FDR = 0.017) [Fig. 1A and Supplementary Table S2 (21)].

In the liver, total PUFAs, n6 fatty acids, and n3 fatty acids were lower in patients with MASH than in those with a normal liver (all FDR < 0.001) and in those with MASH than in those with simple steatosis (FDR = 0.002). In addition, the n6/n3 ratio was higher in those with simple steatosis than in those with a normal liver (FDR = 0.018), and in those with MASH than in those with a normal liver (FDR = 0.014). Gamma-linolenic acid (18:3 n6) was the only PUFA that did not differ between the study groups [Fig. 1A and Supplementary Table S2 (21)].

### SFA Proportions in the Liver Are Increased in Those With MASH

The sum of different SFA proportions in the serum was higher in patients with MASH than in those with a normal liver (FDR < 0.001) and in those with steatosis than in those with a normal liver (FDR = 0.046). Consistent with this, the proportions of myristic acid (14:0), palmitic acid (16:0), and stearic acid (18:0) were higher in patients with MASH than in those with a normal liver (all FDR < 0.001) [Supplementary Fig. S2 (21) and Supplementary Table S2 (21)].

In contrast, SFA proportions in SAT and VAT did not show major differences between the study groups. In contrast to serum samples, the total SFA proportion did not differ between the SAT and VAT groups. However, myristic acid (14:0) levels were significantly lower in patients with MASH than in those with a normal liver in both SAT and VAT (FDR=0.013 and 0.11, respectively) (Supplementary Fig. 2 [21] and Supplementary Table S2 (21)].

Reflecting our findings in serum samples, the sum of different SFA proportions was higher in the livers of patients with simple steatosis (FDR = 0.031) and in those with MASH than in those with a normal liver (FDR < 0.001). Among individual FAs, palmitic acid (16:0) and stearic acid (18:0) proportions were higher in those with MASH than in those with a normal liver (both *P* < .001), which explained the majority of the increase in total SFA proportions. In contrast to other SFAs, pentadecanoic acid (15:0) was lower in patients with MASH than in those with a normal liver (FDR < 0.001) and in patients with MASH than in those with simple steatosis (FDR = 0.045) [Supplementary Fig. S2 (21) and Supplementary Table S2 (21)].

### MUFAs Are Altered Less Than PUFAs and SFAs in Relation to MASH

The sum of the different MUFA proportions (mol%) did not differ significantly between the study groups in the different depots. However, the proportion of oleic acid (18:1 n9) in the serum was lower in patients with MASH than in those with a normal liver (FDR=0.012) [Supplementary Fig. S2 (21) and Supplementary Table S2 (21)].

Vaccenic acid (18:1 n7t) proportions in the SAT and VAT depots were higher in patients with MASH than in those with a normal liver (FDR = 0.012 and FDR = 0.006, respectively). However, the proportion of 11-eicosenoid acid (20:1 n9) in the liver was lower in patients with MASH than in those with a normal liver (FDR < 0.001) and in patients with simple steatosis compared to those with a normal liver (FDR = 0.006) [Supplementary Fig. S2 (21) and Supplementary Table S2 (21)].

### Alterations in Estimated Enzyme Activities Associate With MASH

In serum samples, the estimated SCD1 activity was lower in patients with MASH than in those with normal liver (FDR < 0.001). Elongase activity estimated from the 18:0/16:0 ratio was higher in patients with MASH than in those with normal liver [FDR = 0.002; Fig. 1D and Supplementary Table S2 (21]). Estimated D5D activity was lower in patients with MASH than in those with a normal liver (FDR < 0.001) and in those with simple steatosis than in those with MASH (FDR = 0.004) [Fig. 1B and Supplementary Table S2 (21)].

When comparing the results to those in serum, the elongase activity was similar in VAT, showing lower activity in those with MASH compared to those with a normal liver (FDR = 0.034) and in those with simple steatosis compared to those with a normal liver (FDR = 0.034). There were no significant differences in the estimated enzyme activities in the SAT between the study groups [Fig. 1B and Supplementary Table S2 (21)].

In the liver, SCD1 activity was lower in patients with MASH than in those with normal liver, as determined from the 16:1 n7/16:0 (FDR = 0.001) and 18:1 n7/18:0 ratios (FDR < 0.001). The difference was also significant when comparing patients with MASH to those with simple steatosis (FDR = 0.041). Elongase activity (estimated from the 18:0/16:0 ratio) was lower in patients with MASH than in those with normal liver (FDR = 0.006). The estimated D5D levels were lower in patients with simple steatosis than in those with a normal liver (FDR=0.016) [Fig. 1B and Supplementary Table S2 (21)].

### Decrease of Liver PUFAs in MASH Is Independent of Metabolic and Genetic Risk Factors

Because MASLD is closely linked to metabolic syndrome and several SNPs that increase the risk of developing MASLD and MASH, we constructed linear models adjusted for body mass index, diabetes status, serum glucose, insulin, total and lipoprotein lipids, and several single nucleotide polymorphism (*PNPLA3*, *TM6SF2*, *MBOAT7*) to assess their effect on FA alterations in patients with MASLD. The decrease in total PUFAs and increase in the n6 to n3 PUFA ratio in the liver in those with MASH remained significant in all adjusted models, as well as an increase in estimated elongase levels in the serum in those with MASH. Similar to our primary results, palmitic acid (16:0), stearic acid (18:0), and total SFA proportions increased in the serum and liver samples from patients with MASH in all models [Supplementary Figs. S3-S8 (21)]. We also assessed the associations between FA proportions; estimated enzyme activities; and *PNPLA3*, *TM6SF2* and *MBOAT7* genotypes using linear models [Supplementary Figs. S9-S11 (21)]. However, the *PNPLA3*, *MBOAT7,* and *TM6SF2* genotypes did not explain our results [Supplementary Figs. S9-S11 (21)]. Furthermore, using statins did not explain our results [Supplementary Figs. S12-13 (21)].

### *PPARα* and *PPARγ* Show Divergent Correlations With Hepatic PUFAs

**Figure 2.**
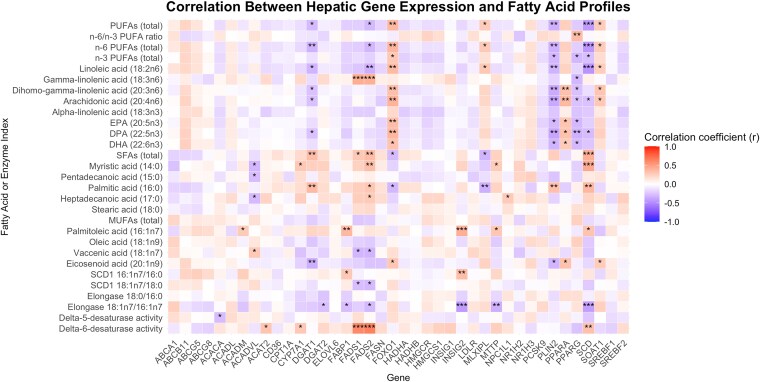
Results of a Spearman's correlation analysis between fatty acid levels and estimated enzyme activities in the liver (y-axis) and gene expression in the liver (x-axis). The intensity of the color indicates the strength of the correlation between variables. Correlations with adjusted *P*-value (FDR) < 0.05 were considered statistically significant and are marked with an asterisk (*). Abbreviations: DHA, docosahexaenoic acid; DPA, docosapentaenoic acid; EPA, eicosapentaenoic acid; FDR, false discovery rate; MUFA, monounsaturated fatty acid; PPARA, peroxisome proliferator-activated receptor α; PPARG, peroxisome proliferator-activated receptor γ; PUFA, polyunsaturated fatty acid; SCD1, stearoyl-CoA desaturase 1; SFA, saturated fatty acid.

To explore potential transcriptional mechanisms underlying the observed differences in tissue fatty acid profiles, we analyzed hepatic gene expression of 40 genes involved in FA desaturation, elongation, transport, storage, and cholesterol metabolism. In the liver, fatty acid desaturase 1 (*FADS1*) and fatty acid desaturase 2 (*FADS2*) expression correlated positively with delta-6-desaturase activity. Stearoyl-CoA desaturase (*SCD*) expression exhibited strong positive correlation with liver total SFAs, myristic acid (14:0) and palmitic acid (16:0), and delta-6-desaturase activity, while correlating negatively with total PUFA levels, n6 PUFA levels, linoleic acid (18:2 n6), arachidonic acid (20:4 n6), docosapentaenoic acid (22:5 n3), and elongase activity (18:1 n7/16:1 n7) (all FDR < 0.05). Forkhead box O1 (*FOXO1*) expression correlated positively with PUFA levels in the liver (all FDR < 0.05), except with gamma-linolenic acid (18:3 n6) and β-linolenic acid (18:3 n3) (Fig. 2).

Liver perlipin-2 (*PLIN2*) expression had a negative correlation with hepatic PUFAs except for gamma-linolenic acid (18:3 n6), α-linolenic acid (18:3 n3), and eicosanoid acid (20:1 n9) and a positive correlation with palmitic acid (16:0) (all FDR < 0.05). Liver peroxisome proliferator-activated receptor α (*PPARα*) expression correlated positively with arachidonic acid (20:4 n6), dihomo-gamma-linolenic acid (20:3 n6), eicosapentaenoic acid (20:5 n3), docosapentaenoic acid (22:5 n3), docosahexaenoic acid (22:6 n3), and eicosanoid acid (20:1 n9) proportions in the liver (all FDR < 0.05). Additionally, transcription factor peroxisome proliferator-activated receptor γ (*PPARγ*) correlated negatively with liver gamma-linolenic acid (18:3 n6), dihomo-gamma-linolenic acid (20:3 n6), arachidonic acid (20:4 n6), eicosapentaenoic acid (20:5 n3), docosapentaenoic acid (22:5 n3), and docosahexaenoic acid (22:6 n3) (all FDR < 0.05). Diacylglycerol acyltransferase 1 (*DGAT1*) demonstrated a similar negative correlation profile with PUFAs than PPARγ. (Fig. 2).

## Discussion

In this study of 183 individuals with severe obesity, we assessed alterations in FA composition in serum, SAT, VAT, and liver depots in relation to MASH. We also estimated SCD1, D5D, D6D, and elongase activities in all depots. We are not aware of any other studies that have comprehensively compared FA composition across liver, serum, and AT depots in patients with MASLD. There were significant differences in the FA proportions in relation to MASLD when comparing the liver and AT depots. Importantly, we observed that although hepatic PUFA proportions were lower in patients with MASH than in those with normal liver, the proportions of arachidonic acid, dihomo-gamma-linolenic acid, and adrenic acid were elevated in both SAT and VAT. Liver SFA increased in patients with MASH compared to those with a normal liver, which was not observed in the AT depots. Furthermore, myristic acid proportions were lower in patients with MASH than in those with a normal liver in both SAT and VAT. Estimated elongase activity increased in the liver and serum but decreased in AT depots in patients with MASH. Our findings raise several pathophysiological hypotheses concerning the role of tissue-specific FA metabolism in MASLD pathogenesis.

Many PUFAs in the liver, including the essential FAs linoleic acid (18:2n6) and α-linolenic acid (18:2n3), were lower in patients with MASH and simple steatosis than in those with a normal liver. However, there were no differences in the proportions of linoleic and α-linolenic acids in AT depots based on liver phenotype, whereas FA proportions in serum samples reflected the trends detected in the liver. Linoleic acid is further metabolized to arachidonic acid, which serves as a substrate for proinflammatory eicosanoid synthesis (32). In this study, the arachidonic acid proportion in the liver was lower in patients with simple steatosis and MASH than in those with normal liver. This indicated that arachidonic acid was used to further synthesize eicosanoids (33). When serum arachidonic acid levels decrease in MASLD, proinflammatory eicosanoids 12-hydroperoxy-eicosatraenoic acid and 12-hydroxyeicosatetraenoic acid levels increase (34). However, this was not supported by the findings of a study using rats (35). Further studies are required to elucidate these changes.

The arachidonic acid proportions in the adipose tissue showed an opposite trend to those in the liver. In SAT and VAT depots, the proportions of dihomo-gamma-linolenic acid, a substrate for arachidonic acid synthesis, arachidonic acid, and adrenic acid were higher in those with MASH than in those with normal liver, and the last 2 FAs differed between those with simple steatosis and those with MASH. As discussed earlier, arachidonic acid can be converted into proinflammatory eicosanoids. AT arachidonic acid levels are an independent risk factor for metabolic syndrome (36). In overweight adults, increased levels of circulating dihomo-gamma-linolenic acid, arachidonic acid, and docosapentaenoic acid have been associated with greater VAT area, which is explained by reduced D5D activity (37). Notably, in addition to proinflammatory FAs, the anti-inflammatory n3 fatty acid docosapentaenoic acid proportion was higher in patients with MASH than in those with a normal liver in VAT. This may be explained by the fact that our VAT samples were collected from the greater omentum, which has anti-inflammatory properties (38). It is intriguing to speculate that docosapentaenoic acid may play a role in controlling inflammatory responses in the omentum.

Notably, we found that the estimated D5D activity was significantly lower in the livers of patients with simple steatosis than in those with normal livers, whereas there were no differences in AT depots. The decrease in PUFAs, including arachidonic acid, could be caused by reduced D5D activity, which has been associated with MASH, independent of obesity (33, 39). Dihomo-gamma-linolenic acid proportions decreased in the livers of patients with MASH but increased in AT and serum depots. Gamma-linolenic acid, a substrate of dihomo-gamma-linolenic acid, was not reduced in any of the deposits in the MASH group. This suggests that although there is sufficient substrate, reduced D5D activity in the liver could cause a shortage of both dihomo-gamma-linolenic acid and arachidonic acid; however, this phenomenon was not observed in AT.

Consistent with our results, a lipidomic analysis of human liver samples showed that the levels of arachidonic acid, eicosapentaenoic acid, and docosahexaenoic acid were lower in patients with MASH than in those with steatosis (33). This suggests that D5D is a rate-limiting enzyme that causes PUFA deficiency in the livers of those with MASH (33). Another possibility is that gamma-linolenic acid is not converted to dihomo-gamma-linolenic acid due to the lack of enzyme elongation of very-long-chain fatty acid protein 5 (ELOVL5). ELOVL5 is involved in the elongation of n-3, n-6 C18, and C20 PUFAs as well as palmitoleic acid (40, 41). It has been shown to be induced during MASH progression in humans, and it has been linked to mitochondrial function in mice and humans as well (42).

We found decreased estimated SCD1 activity in the livers of patients with MASH compared to those with a normal liver. However, there were no significant alterations in SCD1 activity in SAT or VAT depots. SCD1 is the rate-limiting enzyme in MUFA synthesis. MUFAs are the substrates of DNL and can lead to steatosis in the liver (43). SCD1 activity is an important determinant of whether FAs are stored as TGs or are oxidized (43). Previously, both decreased and increased hepatic SCD1 activity have been linked to hepatic steatosis and insulin resistance in obese individuals (16, 44). However, the liver-specific SCD1 knockout resulted in resistance to high carbohydrate diet-induced hepatic steatosis in mice (45). Notably, estimates of enzyme activity based on product-to-precursor ratios differ depending on the plasma fraction analyzed, such as whole plasma, TG, cholesteryl esters, or phospholipids (46, 47). This variability may reflect the same underlying hepatic processes, potentially explaining the inconsistent reports on the estimated SCD1 activity in MASLD. Based on these findings, SCD1 inhibitors have been suggested as potential therapeutic agents against MASLD (48).

Additionally, we found increased elongase activity in the livers of patients with MASH compared to those with normal livers, which has been reported previously (49, 50). Intriguingly, in the VAT samples, the elongase activity behaved oppositely. In the liver, increased elongase activity leads to an increase in long-chain FAs, which can be oxidized, leading to increased levels of reactive oxygen species and oxidative stress (33, 51). Higher estimated elongase activity in the VAT of patients with MASH, but no alterations in SAT, could explain why VAT is considered more metabolically harmful (17). This might also be linked to the fact that adipocytes in VAT tend to grow in size (ie, hypertrophy), which is thought to cause a stronger inflammatory response than growing in numbers (ie, hyperplasia), which is more common in SAT (52, 53).

To better understand the observed alterations in tissue FAs in MASH, we conducted a correlation analysis between FAs and liver gene expression. In the liver, PUFA levels were negatively associated with the expression of key lipogenic and lipid storage-related genes, including *PPARγ*, *PLIN2*, and *SCD*. This pattern is consistent with previous findings that PUFAs, particularly n-3 and n-6 species, suppress hepatic lipogenesis and TG accumulation through transcriptional mechanisms (54, 55). PUFAs have also been shown to inhibit *PPARγ* expression and SCD1 activity, thereby limiting FA desaturation and lipid droplet formation, which may protect against steatosis (56). A similar inverse trend for *DGAT1,* a gene involved in triglyceride synthesis and very low density lipoprotein export, suggests that PUFAs may also influence hepatic lipid export pathways, although the role of *DGAT1* is more complex and context dependent (57, 58). In contrast, *FOXO1*, *PPARα*, and *sterol O-acyltransferase 1 (SOAT1)* expression correlated positively with liver PUFAs. *PPARα* is involved in FA oxidation and the regulation of consuming fat for energy production during fasting (59), *FOXO1* controls enzymes necessary for gluconeogenesis (60, 61), and *SOAT1* participates in cholesterol esterification in the endoplasmic reticulum (62), which may reflect an adaptive regulatory network promoting lipid processing and metabolic flexibility in a PUFA-rich hepatic environment. Further, *PPARα* activates ELOVL5, which participates in elongation of n-3 and n-6 PUFAs as previously discussed (54). Together, these findings highlight that PUFA enrichment in the liver may modulate gene expression toward a less steatotic and more metabolically favorable hepatic function.

It is highly interesting that some patients with severe obesity in this study showed no signs of hepatic steatosis or MASH. These patients consistently exhibited higher hepatic PUFA levels compared to those with MASH, suggesting a potential protective lipid profile in the liver. PUFAs, particularly n-3 fatty acids like eicosapentaenoic acid and docosahexaenoic acid, are also known to enhance mitochondrial β-oxidation, reduce de novo lipogenesis, and exert anti-inflammatory effects via activation of nuclear receptors such as PPARα, while suppressing lipogenic regulators like PPARγ (63).

Our study has some limitations. Samples from different tissue depots and serum samples were not collected from the same patient. However, the characteristics of all these subcohorts based on the depots were similar, and we were able to compare 4 different depots, which have not been reported earlier. In addition, all study participants were severely obese, which might have influenced the results. However, these patients provide a good platform for studying MASLD.

We compared the FA profiles in the liver, serum, visceral, and subcutaneous adipose tissue samples from severely obese patients. We demonstrated that there were significant alterations in the FA profiles between different tissues in relation to MASLD and MASH. In conclusion, we demonstrated a global decrease in hepatic PUFA content, as well as an increase in hepatic SFA content and the n6 to n3 PUFA ratio, in patients with MASH, suggesting increased DNL, whereas in AT depots, levels of arachidonic acid, adrenic acid, and dihomo-gamma-linolenic acid were increased in patients with MASH. Our results suggest that different enzymes are induced in different tissues during MASH development and progression. This requires further study, for example, using transcriptomic data from different tissue depots.

## Data Availability

Some or all datasets generated during and/or analyzed during the current study are not publicly available but are available from the corresponding author on reasonable request.

## References

[1] Younossi ZM, Golabi P, Paik JM, Henry A, Van Dongen C, Henry L. The global epidemiology of nonalcoholic fatty liver disease (NAFLD) and nonalcoholic steatohepatitis (NASH): a systematic review. Hepatology. 2023;77(4):1335‐1347. 36626630 10.1097/HEP.0000000000000004 PMC10026948

[2] Rinella ME, Lazarus JV, Ratziu V, et al A multi-society delphi consensus statement on new fatty liver disease nomenclature. J Hepatol. 2023;79(6):1542‐1556. 37364790 10.1016/j.jhep.2023.06.003

[3] Adams LA, Lymp JF, St Sauver J, et al The natural history of nonalcoholic fatty liver disease: a population-based cohort study. Gastroenterology. 2005;129(1):113‐121. 16012941 10.1053/j.gastro.2005.04.014

[4] Ertle J, Dechêne A, Sowa JP, et al Non-alcoholic fatty liver disease progresses to hepatocellular carcinoma in the absence of apparent cirrhosis. Int J Cancer. 2011;128(10):2436‐2443. 21128245 10.1002/ijc.25797

[5] Donnelly KL, Smith CI, Schwarzenberg SJ, Jessurun J, Boldt MD, Parks EJ. Sources of fatty acids stored in liver and secreted via lipoproteins in patients with nonalcoholic fatty liver disease. J Clin Invest. 2005;115(5):1343‐1351. 15864352 10.1172/JCI23621 PMC1087172

[6] Carli F, Della Pepa G, Sabatini S, Vidal Puig A, Gastaldelli A. Lipid metabolism in MASLD and MASH: from mechanism to the clinic. JHEP Rep. 2024;6(12):101185. 39583092 10.1016/j.jhepr.2024.101185 PMC11582433

[7] Núñez-Sánchez MÁ, Martínez-Sánchez MA, Martínez-Montoro JI, et al Lipidomic analysis reveals alterations in hepatic FA profile associated with MASLD stage in patients with obesity. J Clin Endocrinol Metab. 2024;109(7):1781‐1792. 38217869 10.1210/clinem/dgae028

[8] Mota M, Banini BA, Cazanave SC, Sanyal AJ. Molecular mechanisms of lipotoxicity and glucotoxicity in nonalcoholic fatty liver disease. Metabolism. 2016;65(8):1049‐1061. 26997538 10.1016/j.metabol.2016.02.014 PMC4931958

[9] Puri P, Baillie RA, Wiest MM, et al A lipidomic analysis of nonalcoholic fatty liver disease. Hepatology. 2007;46(4):1081‐1090. 17654743 10.1002/hep.21763

[10] Araya J, Rodrigo R, Videla LA, et al Increase in long-chain polyunsaturated fatty acid n—6/n—3 ratio in relation to hepatic steatosis in patients with non-alcoholic fatty liver disease. Clin Sci Lond Engl. 2004;106(6):635‐643. 10.1042/CS20030326 14720121

[11] Listenberger LL, Han X, Lewis SE, et al Triglyceride accumulation protects against fatty acid-induced lipotoxicity. Proc Natl Acad Sci U S A. 2003;100(6):3077‐3082. 12629214 10.1073/pnas.0630588100 PMC152249

[12] Li ZZ, Berk M, McIntyre TM, Feldstein AE. Hepatic lipid partitioning and liver damage in nonalcoholic fatty liver disease: role of stearoyl-CoA desaturase. J Biol Chem. 2009;284(9):5637‐5644. 19119140 10.1074/jbc.M807616200 PMC2645822

[13] Fridén M, Rosqvist F, Ahlström H, et al Hepatic unsaturated fatty acids are linked to lower degree of fibrosis in non-alcoholic fatty liver disease. Front Med. 2022;8:814951. 10.3389/fmed.2021.814951 PMC8784562 35083257

[14] Rosqvist F, Kullberg J, Ståhlman M, et al Overeating saturated fat promotes fatty liver and ceramides compared with polyunsaturated fat: a randomized trial. J Clin Endocrinol Metab. 2019;104(12):6207‐6219. 31369090 10.1210/jc.2019-00160 PMC6839433

[15] Rosqvist F, Iggman D, Kullberg J, et al Overfeeding polyunsaturated and saturated fat causes distinct effects on liver and visceral fat accumulation in humans. Diabetes. 2014;63(7):2356‐2368. 24550191 10.2337/db13-1622

[16] Kotronen A, Seppänen-Laakso T, Westerbacka J, et al Hepatic stearoyl-CoA desaturase (SCD)-1 activity and diacylglycerol but not ceramide concentrations are increased in the nonalcoholic human fatty liver. Diabetes. 2009;58(1):203‐208. 18952834 10.2337/db08-1074 PMC2606873

[17] Nobarani S, Alaei-Shahmiri F, Aghili R, et al Visceral adipose tissue and non-alcoholic fatty liver disease in patients with type 2 diabetes. Dig Dis Sci. 2022;67(4):1389‐1398. 33788095 10.1007/s10620-021-06953-z

[18] Kotronen A, Seppänen-Laakso T, Westerbacka J, et al Comparison of lipid and fatty acid composition of the liver, subcutaneous and intra-abdominal adipose tissue, and Serum. Obesity. 2010;18(5):937‐944. 19798063 10.1038/oby.2009.326

[19] Fain JN, Madan AK, Hiler ML, Cheema P, Bahouth SW. Comparison of the release of adipokines by adipose tissue, adipose tissue matrix, and adipocytes from visceral and subcutaneous abdominal adipose tissues of obese humans. Endocrinology. 2004;145(5):2273‐2282. 14726444 10.1210/en.2003-1336

[20] Fox CS, Massaro JM, Hoffmann U, et al Abdominal visceral and subcutaneous adipose tissue compartments: association with metabolic risk factors in the framingham heart study. Circulation. 2007;116(1):39‐48. 17576866 10.1161/CIRCULATIONAHA.106.675355

[21] Palomurto S, Virtanen KA, Kärjä V, et al Supplemental Material from Metabolic dysfunction-associated steatotic liver disease alters fatty acid profiles in the liver and adipose tissue. Zenodo. doi:10.5281/ZENODO.15554419. May 30, 2025. PMC12712954 40503664

[22] Männistö VT, Simonen M, Soininen P, et al Lipoprotein subclass metabolism in nonalcoholic steatohepatitis. J Lipid Res. 2014;55(12):2676‐2684. 25344588 10.1194/jlr.P054387 PMC4242459

[23] Trépo E, Valenti L. Update on NAFLD genetics: from new variants to the clinic. J Hepatol. 2020;72(6):1196‐1209. 32145256 10.1016/j.jhep.2020.02.020

[24] Sookoian S, Pirola CJ, Valenti L, Davidson NO. Genetic pathways in nonalcoholic fatty liver disease: insights from systems biology. Hepatology. 2020;72(1):330‐346. 32170962 10.1002/hep.31229 PMC7363530

[25] Brunt EM, Tiniakos DG. Histopathology of nonalcoholic fatty liver disease. World J Gastroenterol. 2010;16(42):5286‐5296. 21072891 10.3748/wjg.v16.i42.5286 PMC2980677

[26] Kleiner DE, Brunt EM, Van Natta M, et al Design and validation of a histological scoring system for nonalcoholic fatty liver disease. Hepatology. 2005;41(6):1313‐1321. 15915461 10.1002/hep.20701

[27] Agren J, Julkunen A, Penttilä I. Rapid separation of serum lipids for fatty acid analysis by a single aminopropyl column. J Lipid Res. 1992;33(12):1871‐1876. 1479296

[28] Venäläinen T, Schwab U, Ågren J, et al Cross-sectional associations of food consumption with plasma fatty acid composition and estimated desaturase activities in Finnish children. Lipids. 2014;49(5):467‐479. 24659110 10.1007/s11745-014-3894-7

[29] Miettinen TA . Cholesterol metabolism during ketoconazole treatment in man. J Lipid Res. 1988;29(1):43‐51. 3356951

[30] Männistö V, Kaminska D, Käkelä P, et al Protein phosphatase 1 regulatory subunit 3B genotype at rs4240624 has a Major effect on gallbladder bile composition. Hepatol Commun. 2021;5(2):244‐257. 33553972 10.1002/hep4.1630 PMC7850313

[31] Benjamini Y, Hochberg Y. Controlling the false discovery rate: a practical and powerful approach to multiple testing. J R Stat Soc Ser B Methodol. 1995;57(1):289‐300.

[32] Levin G, Duffin KL, Obukowicz MG, et al Differential metabolism of dihomo-gamma-linolenic acid and arachidonic acid by cyclo-oxygenase-1 and cyclo-oxygenase-2: implications for cellular synthesis of prostaglandin E1 and prostaglandin E2. Biochem J. 2002;365(2):489‐496. 11939906 10.1042/BJ20011798 PMC1222686

[33] Chiappini F, Coilly A, Kadar H, et al Metabolism dysregulation induces a specific lipid signature of nonalcoholic steatohepatitis in patients. Sci Rep. 2017;7(1):46658. 28436449 10.1038/srep46658 PMC5402394

[34] Xu Y, Han J, Dong J, et al Metabolomics characterizes the effects and mechanisms of quercetin in nonalcoholic fatty liver disease development. Int J Mol Sci. 2019;20(5):1220. 30862046 10.3390/ijms20051220 PMC6429195

[35] Sztolsztener K, Chabowski A, Harasim-Symbor E, Bielawiec P, Konstantynowicz-Nowicka K. Arachidonic acid as an early indicator of inflammation during non-alcoholic fatty liver disease development. Biomolecules. 2020;10(8):1133. 32751983 10.3390/biom10081133 PMC7464179

[36] Williams ES, Baylin A, Campos H. Adipose tissue arachidonic acid and the metabolic syndrome in costa rican adults. Clin Nutr. 2007;26(4):474‐482. 17507118 10.1016/j.clnu.2007.03.004 PMC2730166

[37] Kang M, Lee A, Yoo HJ, et al Association between increased visceral fat area and alterations in plasma fatty acid profile in overweight subjects: a cross-sectional study. Lipids Health Dis. 2017;16(1):248. 29258511 10.1186/s12944-017-0642-z PMC5735636

[38] Meza-Perez S, Randall TD. Immunological functions of the omentum. Trends Immunol. 2017;38(7):526‐536. 28579319 10.1016/j.it.2017.03.002 PMC5812451

[39] Walle P, Takkunen M, Männistö V, et al Fatty acid metabolism is altered in non-alcoholic steatohepatitis independent of obesity. Metabolism. 2016;65(5):655‐666. 27085774 10.1016/j.metabol.2016.01.011

[40] Tripathy S, Jump DB. Elovl5 regulates the mTORC2-Akt-FOXO1 pathway by controlling hepatic cis-vaccenic acid synthesis in diet-induced obese mice [S]. J Lipid Res. 2013;54(1):71‐84. 23099444 10.1194/jlr.M028787 PMC3520542

[41] LEONARD AE, BOBIK EG, DORADO J, et al Cloning of a human cDNA encoding a novel enzyme involved in the elongation of long-chain polyunsaturated fatty acids. Biochem J. 2000;350(3):765‐770. 10970790 PMC1221308

[42] Vouilloz A, Bourgeois T, Diedisheim M, et al Impaired unsaturated fatty acid elongation alters mitochondrial function and accelerates metabolic dysfunction-associated steatohepatitis progression. Metabolism. 2025;162:156051. 39454822 10.1016/j.metabol.2024.156051

[43] Jeyakumar SM, Vajreswari A. Stearoyl-CoA desaturase 1: a potential target for non-alcoholic fatty liver disease?-perspective on emerging experimental evidence. World J Hepatol. 2022;14(1):168‐179. 35126846 10.4254/wjh.v14.i1.168 PMC8790397

[44] Stefan N, Peter A, Cegan A, et al Low hepatic stearoyl-CoA desaturase 1 activity is associated with fatty liver and insulin resistance in obese humans. Diabetologia. 2008;51(4):648‐656. 18286258 10.1007/s00125-008-0938-7

[45] Miyazaki M, Flowers MT, Sampath H, et al Hepatic stearoyl-CoA desaturase-1 deficiency protects mice from carbohydrate-induced adiposity and hepatic steatosis. Cell Metab. 2007;6(6):484‐496. 18054317 10.1016/j.cmet.2007.10.014

[46] Bjermo H, Risérus U. Role of hepatic desaturases in obesity-related metabolic disorders. Curr Opin Clin Nutr Metab Care. 2010;13(6):703‐708. 20823776 10.1097/MCO.0b013e32833ec41b

[47] Peter A, Cegan A, Wagner S, et al Hepatic lipid composition and stearoyl-coenzyme A desaturase 1 mRNA expression can be estimated from plasma VLDL fatty acid ratios. Clin Chem. 2009;55(12):2113‐2120. 19850634 10.1373/clinchem.2009.127274

[48] Sun Q, Xing X, Wang H, et al SCD1 is the critical signaling hub to mediate metabolic diseases: mechanism and the development of its inhibitors. Biomed Pharmacother. 2024;170:115586. 38042113 10.1016/j.biopha.2023.115586

[49] Yamada K, Mizukoshi E, Sunagozaka H, et al Characteristics of hepatic fatty acid compositions in patients with nonalcoholic steatohepatitis. Liver Int. 2015;35(2):582‐590. 25219574 10.1111/liv.12685

[50] Kessler SM, Simon Y, Gemperlein K, et al Fatty acid elongation in non-alcoholic steatohepatitis and hepatocellular carcinoma. Int J Mol Sci. 2014;15(4):5762‐5773. 24714086 10.3390/ijms15045762 PMC4013594

[51] Bradbury MW, Berk PD. Lipid metabolism in hepatic steatosis. Clin Liver Dis. 2004;8(3):639‐671. 15331068 10.1016/j.cld.2004.04.005

[52] Rydén M, Andersson DP, Bergström IB, Arner P. Adipose tissue and metabolic alterations: regional differences in fat cell size and number matter, but differently: a cross-sectional study. J Clin Endocrinol Metab. 2014;99(10):E1870‐E1876. 24937536 10.1210/jc.2014-1526

[53] Tchoukalova YD, Votruba SB, Tchkonia T, Giorgadze N, Kirkland JL, Jensen MD. Regional differences in cellular mechanisms of adipose tissue gain with overfeeding. Proc Natl Acad Sci U S A. 2010;107(42):18226‐18231. 20921416 10.1073/pnas.1005259107 PMC2964201

[54] Jump DB . Fatty acid regulation of hepatic lipid metabolism. Curr Opin Clin Nutr Metab Care. 2011;14(2):115‐120. 21178610 10.1097/MCO.0b013e328342991c PMC3356999

[55] Pawar A, Jump DB. Unsaturated fatty acid regulation of peroxisome proliferator-activated receptor alpha activity in rat primary hepatocytes. J Biol Chem. 2003;278(38):35931‐35939. 12853447 10.1074/jbc.M306238200

[56] Ntambi JM . Regulation of stearoyl-CoA desaturase by polyunsaturated fatty acids and cholesterol. J Lipid Res. 1999;40(9):1549‐1558. 10484602

[57] Chitraju C, Walther TC, Farese RV. The triglyceride synthesis enzymes DGAT1 and DGAT2 have distinct and overlapping functions in adipocytes. J Lipid Res. 2019;60(6):1112‐1120. 30936184 10.1194/jlr.M093112 PMC6547635

[58] Harris CA, Haas JT, Streeper RS, et al DGAT enzymes are required for triacylglycerol synthesis and lipid droplets in adipocytes [S]. J Lipid Res. 2011;52(4):657‐667. 21317108 10.1194/jlr.M013003 PMC3284159

[59] Inagaki T, Dutchak P, Zhao G, et al Endocrine regulation of the fasting response by PPARalpha-mediated induction of fibroblast growth factor 21. Cell Metab. 2007;5(6):415‐425. 17550777 10.1016/j.cmet.2007.05.003

[60] Zhang W, Patil S, Chauhan B, et al Foxo1 regulates multiple metabolic pathways in the liver: effects on gluconeogenic, glycolytic, and lipogenic gene expression. J Biol Chem. 2006;281(15):10105‐10117. 16492665 10.1074/jbc.M600272200

[61] Puigserver P, Rhee J, Donovan J, et al Insulin-regulated hepatic gluconeogenesis through FOXO1-PGC-1alpha interaction. Nature. 2003;423(6939):550‐555. 12754525 10.1038/nature01667

[62] Qian H, Zhao X, Yan R, et al Structural basis for catalysis and substrate specificity of human ACAT1. Nature. 2020;581(7808):333‐338. 32433614 10.1038/s41586-020-2290-0

[63] Pawlak M, Lefebvre P, Staels B. Molecular mechanism of PPARα action and its impact on lipid metabolism, inflammation and fibrosis in non-alcoholic fatty liver disease. J Hepatol. 2015;62(3):720‐733. 25450203 10.1016/j.jhep.2014.10.039

